# Breast cancer patient‐derived scaffolds as a tool to monitor chemotherapy responses in human tumor microenvironments

**DOI:** 10.1002/jcp.30191

**Published:** 2020-12-23

**Authors:** Maria Carmen Leiva, Elena Garre, Anna Gustafsson, Andreas Svanström, Yalda Bogestål, Joakim Håkansson, Anders Ståhlberg, Göran Landberg

**Affiliations:** ^1^ Department of Laboratory Medicine, Sahlgrenska Center for Cancer Research, Institute of Biomedicine, Sahlgrenska Academy University of Gothenburg Gothenburg Sweden; ^2^ Department of Biological Function RISE Research Institutes of Sweden Borås Sweden; ^3^ Wallenberg Center for Molecular and Translational Medicine University of Gothenburg Gothenburg Sweden; ^4^ Department of Clinical Genetics and Genomics Sahlgrenska University Hospital Gothenburg Sweden

**Keywords:** 3D in vitro culture, breast cancer, chemotherapy, decellularized scaffold, extracellular matrix, tumor microenvironment

## Abstract

Breast cancer is a heterogeneous disease where the tumor microenvironment, including extracellular components, plays a crucial role in tumor progression, potentially modulating treatment response. Different approaches have been used to develop three‐dimensional models able to recapitulate the complexity of the extracellular matrix. Here, we use cell‐free patient‐derived scaffolds (PDSs) generated from breast cancer samples that were recellularized with cancer cell lines as an in vivo‐like culture system for drug testing. We show that PDS cultured MCF7 cancer cells increased their resistance against the front‐line chemotherapy drugs 5‐fluorouracil, doxorubicin and paclitaxel in comparison to traditional two‐dimensional cell cultures. The gene expression of the environmentally adapted cancer cells was modulated in different ways depending on the drug and the concentration used. High doses of doxorubicin reduced cancer stem cell features, whereas 5‐fluorouracil increased stemness and decreased the proliferative phenotype. By using PDSs repopulated with other breast cancer cell lines, T‐47D and MDA‐MB‐231, we observed both general and cell line specific drug responses. In summary, PDSs can be used to examine the extracellular matrix influence on cancer drug responses and for testing novel compounds in in vivo‐like microenvironments.

## Graphical abstract

This article introduces a novel 3D in vivo‐like culture system by using decellularized scaffolds from primary breast cancer samples, which allow to study the influence of the tumor microenvironment in response to chemotherapeutic agents.

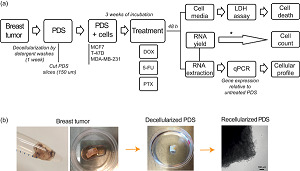

## 1 INTRODUCTION

Breast cancer is a heterogeneous disease with variable phenotypes and genetic features, influencing prognostic, treatment decisions and patient outcome. This heterogeneity has been observed between patients, but also within a tumor, as a response to disease progression and treatments. Furthermore, different phenotypes related to spatial distribution of cells in tissues have been observed, represented by diverse histologic and biochemical properties including variable mutations and expression of biomarkers (Ellsworth et al., 2017). The extracellular matrix (ECM) plays an important role in structural and functional organization of the tumor. The mechano‐chemical stimulation and biological interaction between cells and ECM influence proliferation, survival, migration, invasiveness, and drug response (Frantz et al., 2010; Rijal & Li, 2016; Senthebane et al., 2017). The tumor ECM also promotes survival of the cancer stem cells (CSC), a population that displays stem cell properties, such as self‐renewal, multipotent differentiation and a high tumor‐initiating capacity (Ebben et al., 2010). The CSC phenotype has been linked to poor prognostic features, drug resistance and increased risk of disease recurrences (Akrap et al., 2016; Zhao, 2016).

Traditional two‐dimensional (2D) culture systems used to study breast cancer lack the complexity of three‐dimensional (3D) cell to cell contacts and cell to ECM interactions that occur in vivo. The use of scaffolds is an emerging approach to generate 3D systems able to reflect the in vivo tumor microenvironment characteristics. Scaffolds can be produced using biocompatible and biodegradable synthetic materials like polymers (Balachander et al., 2018; Feng et al., 2013; Ravikrishnan et al., 2016), or using materials derived from natural sources, such as fibrous proteins, biopolymers, or mice‐derived tissue ECM (Dondajewska et al., 2018; Florczyk et al., 2013; Miyauchi et al., 2017; Rijal & Li, 2017). Nevertheless, the selection of the material is crucial due to several factors related to the scaffold composition and structure, such as stiffness, porosity, dimensionality and presence of adhesive proteins, all influencing the behaviors of the attached and growing cancer cells (Rijal & Li, 2016). With the aim to develop clinically relevant scaffolds, the use of human decellularized tissues, especially patient‐derived tumor material is gaining popularity (Hoshiba, 2019; Piccoli et al., 2018; Pinto et al., 2017; Tian et al., 2018). Furthermore, tissues from breast cancer patients have previously been used as growth platforms to study the ECM influence on tumor progression (Jin et al., 2018; Liu et al., 2019).

Recently, we showed that patient‐derived scaffolds (PDSs) produced from decellularized breast cancer tissue retained the natural ECM architecture and composition, representing the complexity of the tumor microenvironment (Landberg et al., 2020). Breast cancer cells that repopulated PDSs were influenced by the microenvironment and were clearly mimicking the in vivo‐like cell–ECM interaction. Typical PDS‐induced cellular features were increased fractions of CSC and epithelial–mesenchymal transition (EMT) cells, accompanied by a decreased proliferation.

In this study, we have analyzed the influence of the PDS microenvironments in response to therapeutic approaches. Three mechanistically different chemotherapeutic drugs were tested, all of them used in the front‐line treatment of breast cancer (Senkus et al., 2015): 5‐fluorouracil (5‐FU), a pyrimidine analog that is transformed into three active metabolites involved in thymidylate synthase inhibition and incorporated into DNA and RNA (Longley et al., 2003); doxorubicin (DOX) which exerts its effect through inhibition of topoisomerase II, though other mechanisms as intercalation into the DNA or free radical formation have been explored (Gewirtz, 1999); paclitaxel (PTX), a taxane with activity in the stabilization of microtubules during the mitosis process, stopping cell division (Weaver, 2014). Three different cancer cell lines representing different breast cancer subtypes, estrogen receptor‐positive MCF7 and T‐47D and estrogen receptor‐negative MDA‐MB‐231, were used to repopulate the PDSs, and gene expression analyses were used to determine the cellular phenotypes of cells enriched by the drug treatments. The results highlight how drug responses are affected by the tumor microenvironment, providing new information compared with the traditional 2D cultures and another 3D model, which might be of clinical importance regarding treatment prediction as well as in the design and evaluation of novel cancer drugs.

## 2 MATERIALS AND METHODS

### 2.1 Cell culture

MCF7, T‐47D, and MDA‐MB‐231 cells were purchased from American Type Culture Collection. MCF7 was cultured in Dulbecco's modified Eagle's medium (DMEM) supplemented with 10% fetal bovine serum, 1% penicillin/streptomycin, 1% *l*‐glutamine (all from Thermo Fisher Scientific) and 1% MEM Non‐Essential Amino Acids (Sigma‐Aldrich). MDA‐MB‐231 and T‐47D were cultured in Roswell Park Memorial Institute 1640 medium supplemented with 10% fetal bovine serum, 1% penicillin/streptomycin, 1% sodium pyruvate, and 1% *l*‐glutamine (Thermo Fisher Scientific). Cells were cultured at 37°C in a 5% CO_2_ humidified atmosphere. Cell media was renewed every 3–4 days and cells were discarded after passage 30.

### 2.2 Tumor decellularization

**Figure 1:**
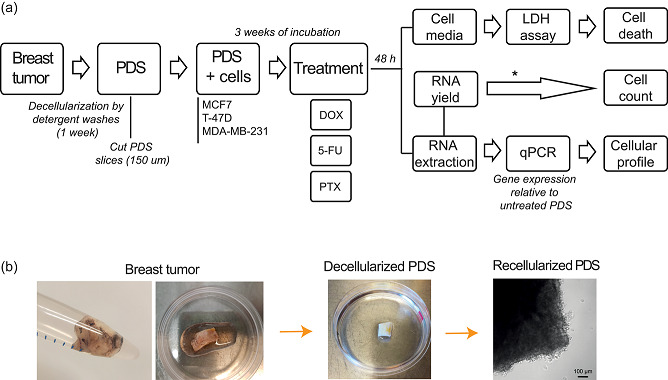
Schematic illustration of the patient‐derived scaffolds (PDSs) production. (a) Workflow for generating PDSs describing the different steps from tumor collection to decellularization, cell culture, treatment and analyses carried out to study drug response. *Indirect measurement. (b) Pictures of breast tumor tissue, a decellularized PDS and a microscopy image of MCF7 cells cultured on PDS. In the contrast‐phase image of a recellularized scaffold, round‐shaped cells can be observed in the border of the PDS. Scale bar = 100 µm. 5‐FU, 5‐fluorouracil; DOX, doxorubicin; LDH, lactate dehydrogenase; PTX, paclitaxel; qPCR, quantitative polymerase chain reaction

Breast cancer primary samples were collected directly after surgery from the Clinical Pathology Diagnostic Unit at the Sahlgrenska University Hospital. Material from 15 patients was used in this study: 13 invasive ductal carcinomas, 1 invasive lobular carcinoma, and 1 in situ carcinoma. The histopathological characteristics of the tumors included in this study are detailed in Table S1. The use of patient material for this project was approved by the Regional Research Ethics Committee (Regionala Etikprövningsnämnden) in Gothenburg (DNR: 515‐12 and T972‐18). All research was performed according to ethical guidelines and informed consent was obtained from all the participants in the study. The decellularization protocol for breast cancer PDSs followed our earlier published protocols (Landberg et al., 2020). In brief, tumors were washed with a lysis buffer composed of 0.1% sodium dodecyl sulfate (SDS), 0.02% Na‐Azide, 5 mM 2H_2_O‐Na_2_‐EDTA, and 0.4 mM phenylmethylsulfonyl fluoride (Sigma‐Aldrich) for 6 h followed by a rinse step in the same buffer without SDS for an additional 15 min. This process was repeated twice, and then, decellularized tumors were washed for 72 h in distilled water which was exchanged every 12 h to remove cell debris followed by 24 h wash in phosphate‐buffered saline (PBS; Medicago). After decellularization (Figure 1), PDSs were cut in 6 mm diameter pieces using a biopsy punch needle, and then sliced in 150 µm slices using a CM3050‐S cryotome (Leica) to get several slices from the same PDS (from 5 up to 60, depending on the tissue). PDS slices were sterilized in peracetic acid 0.1% (Sigma‐Aldrich) for 1 h at room temperature, followed by 24 h washing in PBS + 1% Antibiotic–Antimycotic (Thermo Fisher Scientific), at 37°C and gentle agitation at 175 rpm (Incu‐Shaker^TM^ 10L, Benchmark). PDSs were stored in a buffer containing PBS, 0.02% Na‐Azide, and 5 mM EDTA at 4°C.

### 2.3 Patient‐derived scaffolds recellularization

Before recellularization, PDSs were soaked in cell culture media for 24 h to remove residual storage buffer. PDS slices were placed in a 48 wells‐plate and seeded with 3 × 10^5^ cells in 500 µl of cell line specific media supplemented with Antibiotic‐Antimycotic (1%). After 24 h, the PDSs were transferred to a new plate with fresh media. The PDSs were transferred to new media once or twice a week, up to 21 days of incubation.

### 2.4 Drug treatment

The drugs tested in this article were purchased from Apoteket (Sweden). 5‐FU (50 mg/ml, Accord) and DOX (2 mg/ml, Actavis) were dissolved in a saline solution, whereas PTX (6 mg/ml, Fresenius Kabi) was dissolved in 50% Kolliphor/50% ethanol (K/EtOH). Kolliphor EL was purchased from Sigma Aldrich to assess solvent toxicity.

For treatment of the 2D cultures, about 1.2‐2 × 10^5^ cells were seeded in a six wells‐plate. After 24 h the treatments were administered at the following doses: 5‐FU at 100 µM, 20 and 200 µM for MCF7, MDA‐MB‐231 and T‐47D cells, respectively; DOX at 0.3, 0.3, and 0.2 µM for MCF7, MDA‐MB‐231, and T‐47D cells, respectively; and PTX at 25 nM for MCF7 cells. These concentrations correspond to the IC_50_ of the cell line cultured in 2D calculated with a proliferation assay (Figure S1; Alamar blue; Thermo Fisher Scientific). Cells were incubated with the treatments for 48 h (*n* = 3) before harvesting.

PDS growing cells were treated after 21 days of cell culture in PDSs. Then, PDSs were placed in a 48 wells‐plate with fresh media and treatments for 48 h. The same drug dose used in the 2D cultures and also concentrations 5‐, 10‐, 50‐ 100‐, 500‐, and 1000‐fold higher were tested on the PDSs. PDSs were randomly assigned to the different experiments, three slices derived from three different PDSs were used for every assay as biological replicates, and in each experiment the same PDS was represented in the treatments as well as in the controls.

### 2.5 RNA extraction

Cells in PDS and cells grown in adherent conditions were harvested in 350 µl of RTL buffer (Qiagen) and stored at −80°C. PDSs were homogenized using a stainless steel bead in TissueLyzer II (Qiagen) for 2 × 5 min at 25 Hrz, and centrifuged at full speed for 3 min. RNA extraction was performed using the RNeasy Micro Kit including DNase treatment in a QIAcube machine (all from Qiagen). RNA concentration was measured by NanoDrop (Thermo Fisher Scientific).

### 2.6 Gene expression analysis

Reverse transcription and quantitative polymerase chain reaction (qPCR) were performed similarly to our previous study (Landberg et al., 2020). Complementary DNA synthesis was carried out with a GrandScript cDNA synthesis kit (TATAA Biocenter) in a T100 Thermal Cycler (BioRad) in 20 μl reaction mix at 25°C for 5 min, 42°C for 30 min, 85°C for 5 min followed by cooling to 4°C until subsequent analysis. RNA Spike II (TATAA Biocenter) was previously added to every sample as an RNA stability control. After that, all samples were diluted 1:5 or 1:6 with RNAse free water (Thermo Fisher Scientific). qPCR was performed on a CFX384 Touch Real‐Time PCR Detection System (Bio‐Rad) using 1X SYBR GrandMaster Mix (TATAA Biocenter), 400 nM of each primer (Table S2), and 2 μl diluted complementary DNA in a final reaction volume of 6 μl. The temperature profile was 95°C for 2 min followed by 35‐50 cycles of amplification at 95°C for 5 s, 60°C for 20 s, and 70°C for 20 s and a melting curve analysis at 65°C to 95°C with 0.5°C/s increments. Cycle of quantification values were determined by the second derivative maximum method with the CFX Manager Software version 3.1 (Bio‐Rad). Gene expression was normalized using reference genes identified with the NormFinder algorithm and expressed as relative quantities (log2) to the untreated 2D cells or to untreated PDSs, depending on the analysis.

Data preprocessing was performed using GenEx (MultiD). PCA plots and heatmaps were calculated using GenEx, and autoscaled data was represented. The radar charts illustrating the average of several genes were calculated using Excel 2016. All experiments were conducted in accordance with the Minimum Information for Publication of Quantitative Real‐Time PCR Experiments guidelines (Bustin et al., 2009).

### 2.7 Cytotoxicity assay

Cell death was assessed using lactate dehydrogenase (LDH) assay (Roche) on conditioned media from PDS cultures. At the same time of harvesting cells and PDSs for qPCR analysis, cell media was collected and stored at 4°C, following the manufacturer's recommendations. A volume of 100 µl of media was added to a 96‐wells plate together with 100 µl of the reaction mix. After 30 min of incubation absorbance was measured at 490 nm in a Multi‐mode reader (Biotek), using Gen5 software (Biotek). Reference wavelength at 680 nm was subtracted, as well as background signal from the cell media. Data was represented as relative absorbance to the untreated samples.

### 2.8 3D‐printed scaffolds synthesis, cell culture and treatment

For the 3D‐printed scaffold (3DPS) synthesis, alginate of 8% (wt/vol %; Protanal LF 10/60; FMC) and 5% hydroxyapatite (wt/vol %; Sigma‐Aldrich) were mixed in water (Synergy Elix 15; Merck) using an Ultra‐Thurrax T50 digital dispenser (IKA), equipped with an S 25N‐25G dispensing tool, at 5000 rpm overnight and printed in four layers (⌀20 × 2 mm; grid distance 1.5 mm, 90°) using a bioplotter (Envisiontech) equipped with 400 µm extrusion needles. Each layer was cross‐linked in 0.1 M CaCl_2_ (VWR) and ready prints were stored in 0.1 M CaCl_2_ (VWR) at 4°C for up to 2 weeks.

For cell culture, 3DPS were washed in cell culture media, placed in 24 wells‐plates and seeded with 3 × 10^5^ MCF7 cells in supplemented DMEM. After 24 h, 3DPS were moved to a six well‐plates with fresh media, and this process was repeated every 4 days to a total culturing time of 21 days. After that, 3DPS were treated with 5‐FU and DOX at the 10X concentration as the PDS treatments. For RNA extraction, 3DPS were washed twice in cell media, lysed in 700 µl QIAzol (Qiagen) and homogenized for 2 × 2.5 min at 25 Hz. Automated isolation of total RNA from lysates was performed in a QIAcube machine (Qiagen) using a RNeasy Micro Kit (Qiagen) with QIAzol extraction directives. Reverse transcription and qPCR were performed as explained above.

### 2.9 Statistics

Data was processed using GraphPad Prism v7.04 and Excel 2016. Student's *t* test corrected by the Holm–Sidak method was used for comparing two groups and two‐way analysis of variance with Tukey correction was used for comparing more than two groups. *p* < .05 were considered significant. All experiments were carried out in triplicates unless it was specified, using slices form three different patients in the case of the PDSs, and error bars represent standard deviation of the mean.

## 3 RESULTS

### 3.1 PDSs modulate cellular phenotypes by increasing cancer stem cell features and decreasing proliferation

**Figure 2:**
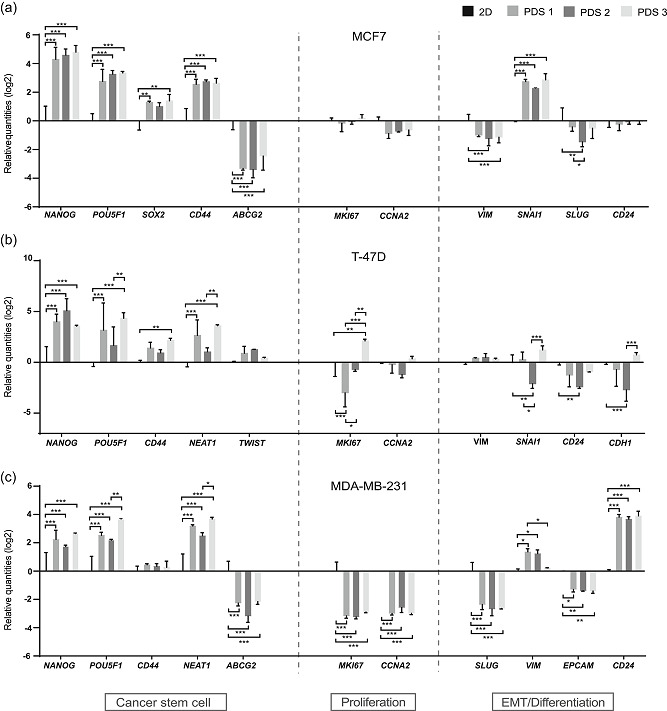
PDSs modulate gene expression enhancing the cancer stem cells low proliferative phenotype. Analysis of gene expression modulation in (a) MCF7, (b) T‐47D, and (c) MDA‐MB‐231 cell lines cultured on PDSs. Data is represented relative to 2D cultures of the same cell line. Mean ± *SD* is shown, *n* = 3. Data from 2 to 3 replicates of each PDS were averaged before analysis. Significant differences between cells growing in PDSs and 2D, as wells as differences between the PDSs from different patients are stated (*p < .05, **p < .01, and ***p < .001). PDS, patient‐derived scaffold

To determine the general PDS effect, we quantified the regulation of marker genes when cancer cells where cultured in PDSs compared to 2D cultures. We quantified the regulation of a set of markers for proliferation (*MKI67*, *CCNA2*), pluripotency and CSC (*NANOG, POU5F1*, *SOX2*, *CD44*, *TWIST*, *NEAT1*, *ABCG2*), EMT (*VIM*, *SNAI1*, *SLUG*), and differentiation (*CDH1*, *EPCAM*, *CD24*). For this purpose, two luminal (MCF7 and T‐47D) and one basal (MDA‐MB‐231) breast cancer cell lines, representing different tumor subtypes were used (Figure 2).

Here, all three cell lines cultured in PDSs were shown to have a consistent upregulation of CSC markers, except for *ABCG2*, which was significantly downregulated in the PDSs cultured with MCF7 (Figure 2a) and MDA‐MB‐231 (Figure 2c), and expression was not detected in the PDSs cultured with T‐47D. A trend towards a downregulation of proliferation markers in comparison to 2D cultures was also observed, with a stronger modulation in MDA‐MB‐231 cells (Figure 2c), and a high variability in the expression of *MKI67* between the different scaffolds cultured with T‐47D (Figure 2b). Furthermore, markers for EMT and differentiation varied substantially between the cell lines in response to the PDS environment. As an example, the EMT marker *VIM* was downregulated in the PDSs cultured with MCF7, upregulated with MDA‐MB‐231 and non‐altered with T‐47D, in comparison to the 2D cultured cell lines. Our results indicate that PDSs may induce a CSC enriched and low proliferative phenotype, supporting previous data published by our group (Landberg et al., 2020).

### 3.2 PDSs increase cancer cell resistance against chemotherapy compounds

Next, MCF7 cells cultured on PDSs and in 2D were treated with the chemotherapy agents 5‐FU, DOX, and PTX at the established IC_50_, a concentration that reduced proliferation by 50% in cells growing in 2D cultures (Figure S1). After 48 h of treatment, cell media was collected and LDH assay was performed to determine the cell death ratio. Cells were harvested from the PDSs and RNA was extracted for gene expression analyses by qPCR as well as for an indirect measurement of the cell count by the total RNA yields (Figure 1a).

**Figure 3:**
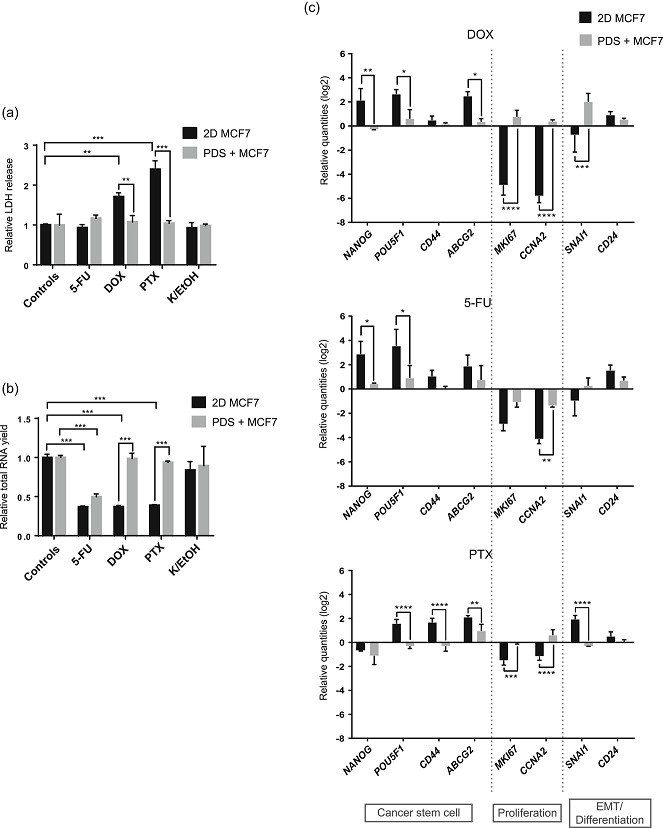
Cells cultured in PDSs present a higher resistance against chemotherapy drugs than 2D cultures. Measurement of (a) cell death, (b) total RNA yield and (c) gene expression in MCF7 cells grown in PDSs and 2D culture following treatment with 5‐fluorouracil (5‐FU), doxorubicin (DOX), and paclitaxel (PTX). Data is normalized to untreated 2D or PDSs, respectively. Mean ± *SD* is shown, *n* = 3. Significant differences between 2D and PDS or between untreated controls and treatments are represented (*p < .05, **p < .01, and ***p < .001). PDS, patient‐derived scaffold

For the 2D cultures, increased cell death and decreased total RNA yield were observed for DOX and PTX treatments (Figure 3a,b), whereas these treatments did not induce cell death or decrease the total RNA yield when administered to cells cultured in the PDS model. There was no effect on cell death or proliferation of the PTX solvent alone (kolliphor and ethanol ‐ K/EtOH) in 2D or PDS cultures. In the case of 5‐FU, similar responses were observed in PDS and 2D growth models, with no cell death measured by LDH but with a decrease in the total RNA yield. These results indicated an increased resistance for two out of three chemotherapeutic compounds in the PDS system compared to 2D cultures. Furthermore, all three drugs triggered more pronounced gene expression changes in the cells cultured in 2D compared to PDSs, illustrated by a general upregulation of CSC associated markers and a decrease in proliferation genes in 2D cultures (Figure 3c). DOX and PTX treatments showed larger differences between the drug response in 2D and PDS cultures than 5‐FU, supporting cell death and total RNA yield results. Similar to the basal response to PDSs without treatment (Figure 2), diverse response patterns were observed between the EMT/differentiation markers when treated with different chemotherapy compounds. Taken together, these results show that gene expression modulation after drug treatments is significantly higher in 2D compared to PDS cultures, supporting a context‐dependent drug response.

### 3.3 Different doxorubicin concentrations led to cell phenotype selections in PDSs growing cells

We next analyzed the cellular response by gene expression analyses of MCF7 cells growing in PDSs exposed to increasing concentrations of DOX up to 100‐fold the IC_50_ defined in 2D cultures. In addition, quantifications of cell death and total RNA levels were used as complementary measurement of the drug effect.

**Figure 4:**
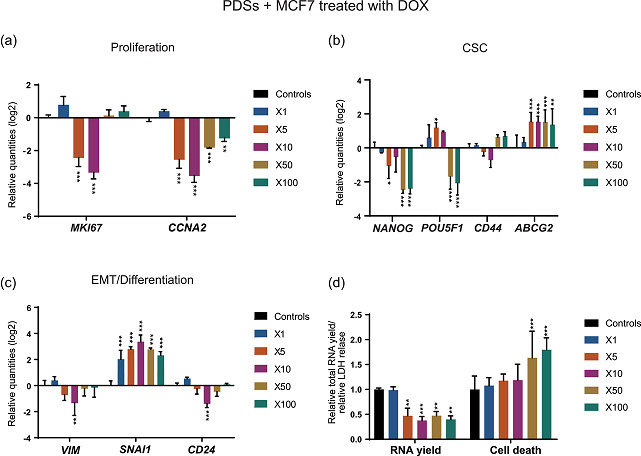
Treatment with DOX produces concentration‐based effects in PDSs cultured with MCF7. Modulation of the expression of genes related to (a) proliferation, (b) cancer stem cells (CSC), and (c) epithelial–mesenchymal transition (EMT)/differentiation at increasing concentrations of DOX indicated as fold‐change respect to the IC_50_ in 2D cultures. Data is relative to gene expression in untreated PDSs (log2). (d) DOX effect on the total RNA yield and LDH release, as surrogate measurements for cell count and cell death, respectively, quantified at the same drug concentrations. Relative quantities to untreated PDSs are represented. Mean ± *SD* is shown, *n* = 3. Significant differences to the untreated controls are stated (*p < .05, **p < .01, and ***p < .001). DOX, doxorubicin; LDH, lactate dehydrogenase; PDS, patient‐derived scaffold

For drug concentrations up to 10‐fold the IC_50_ value, there was a significant decrease in the expression of proliferation markers, whereas only minor effects were observed in the CSC‐related genes relative to untreated PDS cultures (Figure 4a,b). However, when the concentration was increased to 50‐ and 100‐fold, a clear switch in the expression pattern was observed. The expression of proliferation genes became more similar to the untreated PDS cultures whereas the CSC‐related markers *NANOG* and *POU5F1* were significantly downregulated. On the contrary to other CSC‐related markers, the gene *ABCG2*, which encodes for a drug transporter, was significantly upregulated from the 5X drug concentration (Figure 4b), suggesting an enhancement of this drug resistance mechanism. Regarding EMT/differentiation, diverse responses were shown in between the markers. A similar behavior as for the proliferation markers was observed for *VIM* and *CD24*, with a switch in the expression pattern from a significant downregulation at the concentration 10X to a similar response as the untreated PDS cultures when the concentrations were increased (Figure 4c). In contrast, there was a clear increased expression of *SNAI1* for all tested concentrations in PDS cultures.

Quantification of total RNA levels revealed a decrease in yield in the PDS cultures treated with DOX from the concentration 5X relative to untreated controls, mirroring the proliferation decrease observed in the gene expression analysis (Figure 4d). However, an increase in cell death was not detected until the drug concentrations were increased up to 50‐fold. The increased cell death ratio at the highest DOX concentrations correlates with the switch in the gene expression phenotype, suggesting a substantial different drug effect and a potential selection of cell populations at this high concentration.

These findings present two different cellular phenotypes in PDS growing MCF7 following DOX treatment: a low proliferative population at the intermediate concentrations (5X and 10X) that switches towards a recovery of the basal proliferative state while decreasing the CSC phenotype at the highest doses (50X and 100X). Although the effect at intermediate doses resembles the drug response in 2D cultured cells at the IC_50_ concentration (Figure 3c), higher doses resulted in a completely different response in the PDS cultures.

Additionally, the drug response by cells cultured in PDSs was compared to cells cultured in a 3DPS (Figure S2). Here, heatmap analysis shows significant differences in the expression of the CSC markers (*POU5F1* and *CD44)* and EMT markers (*SNAI1* and *VIM)* between PDS and 3DPS cultured cells at 10X DOX concentration.

### 3.4 Treatment with 5‐fluorouracil enriches for a low proliferative CSC phenotype in cells cultured in PDSs

**Figure 5:**
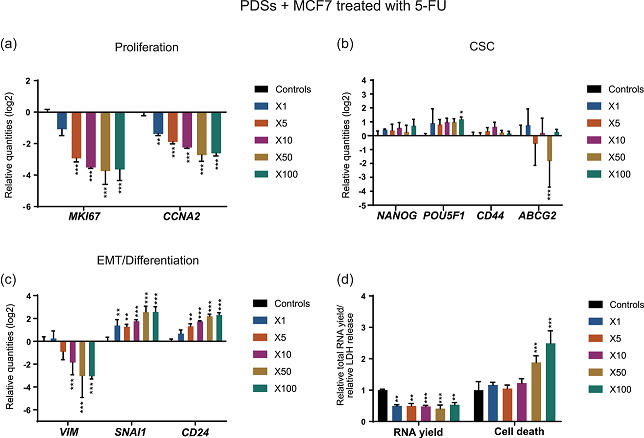
Treatment with 5‐FU enhances the CSC low proliferative phenotype in PDSs cultured with MCF7 cells. Modulation of the expression of genes related to (a) proliferation, (b) CSC and (c) EMT/differentiation at increasing concentrations of 5‐FU indicated as fold‐change respect to the IC_50_ in 2D cultures. Data is relative to gene expression in untreated PDSs (log2). (d) 5‐FU effect on the total RNA yield and LDH release, as surrogate measurements for cell count and cell death, respectively, quantified at the same drug concentrations. Relative quantities to untreated PDSs are represented. Mean ± *SD* is shown, *n* = 3. Significant differences to the untreated controls are stated (*p < .05, **p < .01, and ***p < .001). 5‐FU, 5‐fluorouracil; CSC, cancer stem cell; EMT, epithelial–mesenchymal transition; LDH, lactate dehydrogenase; PDS, patient‐derived scaffold

MCF7 cells cultured in PDSs were treated with increasing 5‐FU concentrations, and changes in gene expression were monitored (Figure 5). The expression of proliferation genes was downregulated in a dose‐dependent manner in low 5‐FU concentration (1X–10X), indicating a plateau at higher drug concentrations (50X and 100X; Figure 5a). This antiproliferative effect upon drug treatment was also indirectly observed at the lowest concentration of 5‐FU tested, by a reduction of total RNA levels (Figure 5d).

In contrast to DOX, 5‐FU enhanced the expression of CSC‐related genes (Figure 5b), although this upregulation was only significant for *POU5F1* at the highest concentration. Furthermore, 5‐FU did not induce *ABCG2* expression, on the contrary, a significant downregulation was observed at the 50X drug concentration. In addition, there was no switch in gene expression modulation as observed with an increasing cell death ratio for DOX treatment at 50‐ and 100‐fold higher drug concentrations (Figure 5d). Differentiation and EMT markers showed a dose‐dependent gene expression modulation, following the general trend observed for proliferation‐related genes. Similar to DOX treatment, a decrease in the expression of *VIM* and an increase in *SNAI1* were observed after 5‐FU treatment. In contrast, *CD24* was significantly upregulated (Figure 5c).

In general, treatment with 5‐FU of MCF7 cells growing in PDSs enriched for the CSC phenotype as indicated by a slightly increased expression of CSC markers and a decreased expression of proliferation markers. In contrast to DOX, 5‐FU caused similar drug‐induced gene expression patterns in both 2D and PDS cultures when using higher drug concentrations in the 3D model. The responses were nevertheless more pronounced in the 2D cultures and this was especially noticeable for the CSC‐related markers (Figure 3c). In comparison to 3DPS cultured cells, PDS cultured cells treated with 5‐FU were shown to have a different response in the expression of CSC (*NANOG* and *POU5F1*) and EMT (*SNAI1*) markers, similar to the results observed with DOX (Figure S2).

### 3.5 Cells cultured in PDSs show a higher resistance against paclitaxel treatment

**Figure 6:**
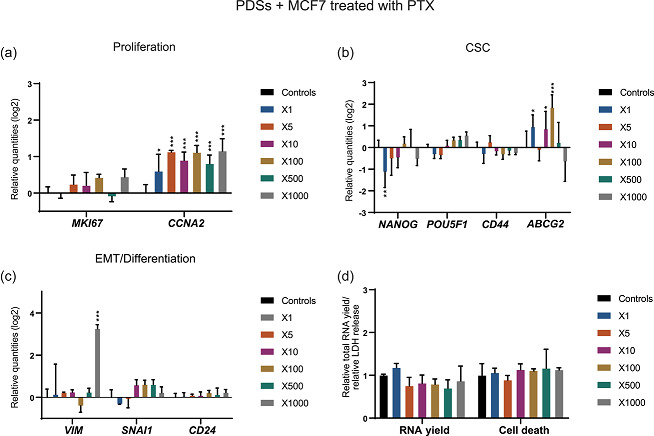
Treatment with PTX caused minor effects on MCF7 cells cultured in PDSs. Modulation of the expression of genes related to (a) proliferation, (b) CSC and (c) EMT/differentiation at increasing concentrations of PTX indicated as fold‐change respect to the IC_50_ in 2D cultures. Data is relative to gene expression in untreated PDSs (log2). (d) PTX effect on the total RNA yield and LDH release, as surrogate measurements for cell count and cell death, respectively, quantified at the same drug concentrations. Relative quantities to PDSs treated with an equivalent dose of PTX solvent (Kolliphor/ethanol) are represented. The solvent toxicity is illustrated in Figure S2. Mean ± *SD* is shown, *n* = 3. Significant differences to controls are stated (*p < .05, **p < .01, and ***p < .001). CSC, cancer stem cell; EMT, epithelial–mesenchymal transition; LDH, lactate dehydrogenase; PDS, patient‐derived scaffold; PTX, paclitaxel

**Figure 7:**
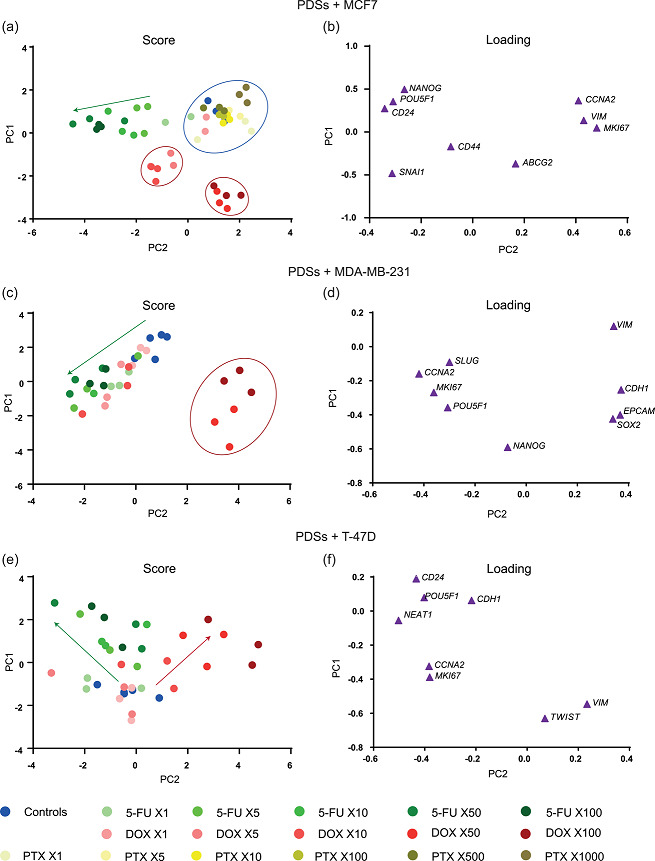
Gene expression profiles of increasing drug concentrations in three different cell lines cultured in PDSs. Principle component analysis (PCA) representation of the gene expression profile of (a) MCF7, (c) MDA‐MB‐231 and (e) T‐47D cells growing in PDSs, following treatment with 5‐FU, DOX, and PTX (only in MCF7) and compared to untreated PDSs (controls). Each dot represents a PDS sample, and three different PDSs are included per treatment. Drug concentrations are calculated as fold‐change respect to the IC_50_ in 2D cultures. Circles represent separated clusters and arrows show the trend at increasing drug concentrations. (b,d, and f) PCA genes loading illustrate the contribution to the PCA scores in a,c and e, respectively. Represented data is autoscaled. 5‐FU, 5‐fluorouracil; DOX, doxorubicin; PDS, patient‐derived scaffold; PTX, paclitaxel

The effect of increased concentrations of PTX was also studied in PDS growing MCF7. For these experiments, we increased the drug concentration up to 1000‐fold the IC_50_, but little to no effect was detected in the cells cultured in PDSs (Figure 6). When the modulation of specific genes was analyzed in detail, only changes in a few marker genes were observed following treatment with PTX. Differing from the other treatments, there was an increased expression of the proliferation marker *CCNA2* at all the doses analyzed (Figure 6a). Other changes in gene expression after PTX treatment were related to the upregulation of *ABCG2* at various doses (Figure 6b) and the upregulation of *VIM* at the highest concentration used (Figure 6c). As illustrated in the Principle Component Analysis (PCA) plots, the PDS cultured cells treated with PTX clustered together with untreated PDSs and PDSs treated with the lowest concentrations of DOX and 5‐FU (Figure 7a), corroborating the lack of effect on gene expression modulation upon PTX treatment. Furthermore, no effect on the total RNA levels or cell death ratio was observed with this drug (Figure 6d). To exclude PTX solvent toxicity, PTX effect was normalized to the effect of K/EtOH, since high solvent toxicity was detected using the 500X concentration (Figure S3). Importantly, the minor changes in cell death ratio, total RNA levels and gene expression modulation in PDS cultured MCF7 cells upon PTX treatment contrast the pronounced effects observed in 2D cultures (Figure 3). Thus, the presented data clearly indicates that PDS microenvironments profoundly affect the cancer cell response to PTX treatment.

### 3.6 Repopulation of PDSs with different breast cancer cell lines presents a variety of expression profiles following treatment with chemotherapy compounds

To evaluate cell line‐dependent responses to treatments, PDSs repopulated with MDA‐MB‐231 or T‐47D cells were treated with DOX or 5‐FU as previously described (Figure 1, Figure S1), but PTX was omitted due to the lack of effect in the previous analyses.

As mentioned above, DOX treatment of MCF7 cells in PDSs produced two different gene expression patterns related to the drug concentration, whereas there was a gradual transition in expression changes from untreated PDSs to higher concentrations of 5‐FU (Figure 7a,b). MDA‐MB‐231 cells grown in PDSs showed a similar behavior as MCF7, grouping in two different clusters after DOX treatments associated again with the highest concentrations (50X and 100X) where cell death was increased (Figure 7c,d; Figure S4). In contrast, there was a notably different drug‐related response in PDSs cultured with T‐47D cells, with no separate clusters but instead a gradual change in the expression profile from the untreated PDSs to increasing doses in both chemotherapy treatments (Figure 7e,f). Moreover, the variability in the drug response between the different PDSs was especially noticeable for this cell line. This difference was illustrated in the PCA plot where the dots for individual PDSs were highly spread, representing the cellular phenotype diversity in response to the treatment with 5‐FU and DOX for T‐47D samples (Figure 7e).

**Figure 8:**
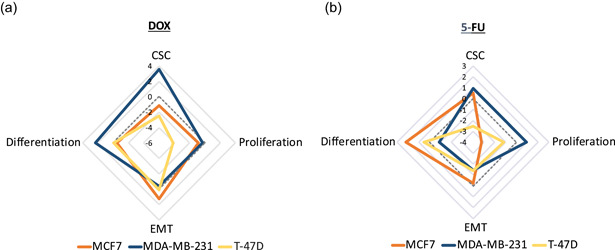
Comparison of the drug responses in PDSs seeded with three different cell lines. Radar charts represent the average modulation of genes related to CSC, proliferation, EMT, and differentiation following treatment with (a) DOX and (b) 5‐FU at the concentration 50X. Data represent relative quantities to untreated PDSs cultured with the specific cell line (MCF7, MDA‐MB‐231, or T‐47D) in log2 scale (*n* = 3). The genes used for the average calculation were chosen based on their relevance in the drug response for every model and are detailed in Table S3. 5‐FU, 5‐fluorouracil; CSC, cancer stem cell; DOX, doxorubicin; EMT, epithelial–mesenchymal transition; PDS, patient‐derived scaffold

Despite the similarities and discrepancies in the general pattern of gene expression changes upon drug treatments, the individual genes seemed to differ in a cell line dependent manner where the cellular phenotypes were more similar in the two luminal cell lines, MCF7 and T‐47D. When comparing the three cell lines responses to DOX treatment using a concentration (50X) that produced clear differences between the treatments in PDSs (Figure 8a), T‐47D and MCF7 cells were characterized by a downregulation in CSC and proliferation genes, and these changes were most pronounced in T‐47D. In contrast, MDA‐MB‐231 cells showed a marked increase in the expression of CSC and differentiation‐related genes. The pronounced upregulation of *SOX2* was the main modulation among the CSC markers in MDA‐MB‐231 (Figure S4). On the other hand, 50X 5‐FU treatment using PDSs populated with MCF7 and T‐47D cells produced cancer cells with an enrichment of the differentiated phenotype, as indicated by the increased expression of differentiation markers, together with the decrease in proliferation genes expression (Figure 8b). Regarding CSC‐related genes, no similarities were observed between the estrogen‐responsive cell lines (MCF7 and T‐47D), since PDS growing MCF7 and MDA‐MB‐231cells showed a slightly elevated expression, whereas a clear decrease was observed with T‐47D cells (Figure S5).

When comparing drug responses between 2D and PDSs cultured T‐47D and MDA‐MB‐231 cells at the IC_50_ (2D) drug concentration, there were no clear differences between the two growth systems as previously observed for MCF7. For MDA‐MB‐231 cells there was a significant reduction in the total RNA levels following 5‐FU and DOX treatments in 2D in comparison to cells growing in PDSs, but there were no differences in gene expression (Figure S4). On the other hand, in T‐47D cells we observed an enhanced drug response and gene expression modulation in 2D compared to PDSs, whereas this difference diminished with increasing drug concentrations in the PDS cultures (Figure S5).

Taken together, our findings analyzing three different cell lines demonstrate that the microenvironment provided by the PDSs indeed influences drug response, and that the cell line selection could influence the specific gene expression fingerprint for chemotherapeutic therapies.

## 4 DISCUSSION

This study describes the use of breast cancer PDSs as a physiologically relevant and in vivo‐based 3D model to study the influence of the tumor microenvironment in chemotherapy response. Even though most cancer studies are based on analyses of tumor cells, the microenvironment has been suggested to substantially influence tumor progression and malignancy (Lu et al., 2012). Therefore, it is relevant to include the microenvironment in growth models assessing therapy responses to optimally mimic human‐like conditions and relevant modeling of why some patients respond to a treatment whereas others are unaffected.

The PDS‐platform used in this article was recently developed in our laboratory in an attempt to monitor how the microenvironment will influence cancer cells adapting to a decellurarized tumor scaffold. Earlier published data supports that PDSs keep similar characteristics to in vivo tumors with links between the composition of the scaffolds and clinical properties (Landberg et al., 2020). Interestingly, PDSs also retained exosome‐related proteins secreted by other stroma components, supporting that the presented PDS model indeed mirrors the complex microenvironment formed by various cell types (Landberg et al., 2020). The gene expression data showed here using untreated PDSs further corroborate and extend the information of processes that are modulated through cell‐ECM interactions, including information from an additional luminal breast cancer cell line, T‐47D. Furthermore, the data obtained from the 3D based PDS model, using various cancer cell lines and treatments, supports that this platform can be suitable for large scale monitoring of comprehensive patient cohorts.

The effect of three front‐line chemotherapeutic compounds on cancer cells cultured in PDSs was assessed using gene panels to determine coordinated drug treatment effects on several tumor biological processes. In line with previous reports, the results clearly indicated a higher drug resistance in cells cultured in PDSs in comparison to the 2D cultures, demonstrating that the growth of cells in a 3D conformation increases their robustness against chemotherapeutic drugs (Miyauchi et al., 2017; Rijal & Li, 2017). As examples, multicellular tumor spheroids showed increased resistance towards PTX and DOX treatments (Imamura et al., 2015; Reynolds et al., 2017) and Hakanson et al. (2011) observed less sensitivity to PTX in MCF7 cells cultured in 3D microwells or fibronectin matrices compared to 2D cultures. Further, breast cancer cells growing in silk scaffolds required a 40‐fold higher DOX dose compared to 2D cell cultures to achieve comparable effects (Dondajewska et al., 2018) and cells cultured in scaffolds from decellularized breast cancer tissues have also shown less induced toxicity in response to 5‐FU treatment (Liu et al., 2019). Other studies using colorectal cancer PDSs have shown similar results, with an increased resistance to 5‐FU in comparison to 2D cultures (D'Angelo et al., 2020; Sensi et al., 2020). However, contradictory drug responses have also been reported when using different 3D models (Hakanson et al., 2011; Hongisto et al., 2013). For instance, Hongisto et al. (2013) compared the response to 102 compounds in cells cultured in two different 3D models, and cells cultured in matrigel were more sensitive to the treatments compared to cells grown in poly(2‐hydroxyethyl methacrylate), having a similar response as the 2D model. We have also observed significant differences in drug response between cells cultured in PDSs and 3DPS, supporting that the effects observed in PDSs are not only influenced by a 3D cell conformation. An advantage of our PDS respect to the 3DPS and other 3D systems is that the scaffolds are derived from human tumors and represent the natural composition and structure of the breast cancer ECM. The higher drug resistance observed in cells growing in PDSs may be associated with the changes in the environmentally adapted cell phenotypes showing increased stemness. Furthermore, we observed an upregulation of *SNAI1* in the untreated PDSs seeded with MCF7, and an aberrant expression of *SNAIL (SNAI1)* and *SLUG* in MCF7 cells has been associated to increased resistance to DOX (Kajita et al., 2004; W. Li et al., 2011). Besides, ECM proteins may form a barrier preventing the drug availability to the cancer cells, and some protein constituents of our PDSs as fibronectins, collagens and laminins have earlier been linked to cell adhesion‐mediated drug resistance (Landberg et al., 2020; Meads et al., 2009; Senthebane et al., 2017).

It is generally accepted that CSC have inherent resistance against chemotherapies in comparison to proliferative populations, resulting in CSC enrichment during treatments (Dean et al., 2005; X. Li et al., 2008). In fact, MCF7 derived CSC have shown more resistance to DOX than MCF7 cells at concentrations up to 1 µM (Yenigun et al., 2013). Furthermore, CSC enrichment has been observed in patients receiving neo‐adjuvant chemotherapy based on docetaxel or DOX and cyclophosphamide at standard doses (X. Li et al., 2008). In this PDS‐based study, DOX treatment at low to intermediate concentrations (0.3–3 µM) showed little effect on CSC markers, but with a clear inhibition of CSC features after treatment at doses above 15 µM (50X). The drug concentrations used in this study were higher compared to concentrations normally used in vitro due to the low sensitivity to chemotherapy compounds observed in PDS cultures. However, the concentrations were within the range of clinically relevant single intravenous administration doses, supporting that PDS treatments indeed mirrored in vivo‐ and human‐like conditions (Liston & Davis, 2017). It can further be hypothesized that the reversion of the antiproliferative effect at the highest doses of DOX together with the reduction in CSC markers may be a consequence of DOX targeting the specific subpopulation with CSC phenotype. In addition, the increased expression of *ABCG2* following treatment with DOX, could be in accordance with the multidrug resistance mechanism of this drug transporter (Stacy et al., 2013).

In the case of 5‐FU treatment using PDSs, a drop in total RNA yield was observed for the lowest concentrations, which was accompanied by a decrease in proliferation as indicated by the downregulation of proliferation genes. To actually increase cell death, 5‐ and 50‐ fold higher doses of 5‐FU were needed for MDA‐MB‐231 and MCF7 cells, respectively. This dual effect of 5‐FU has previously been reported and seems to be related to its mechanism of action, though the mechanism which switches from cytostasis to apoptosis is not completely understood (Hernández‐Vargas et al., 2006). Moreover, there was a slight upregulation of CSC markers after 5‐FU treatment, which is in line with other reports linking CSC to 5‐FU resistance (Dean et al., 2005; Lü et al., 2011; Saha et al., 2016). However, a study by Liu et al. (Liu et al., 2019) using breast cancer decellularized scaffolds repopulated with MCF7 cells described a significant decrease in CSC markers as *POU5F1*, *SOX2*, and *CD49F* after treatment with 5‐FU. These findings differ from the results presented in this article, but in their case similar results have been reported for 2D cultures.

When testing PTX treatment using PDS cultured cells, we observed little to no effects in cell death ratio, total RNA yield or gene expression at concentrations 500‐ and 1,000‐ fold higher than effective IC_50_ concentrations in 2D cultures. Similarly to our observations, a lack of general effects on CSC markers following treatment with PTX has also been reported in spheroids (Reynolds et al., 2017).

The heterogeneous expression of differentiation and EMT markers observed in the PDS cultures before and after treatment seems to be characteristic of the tumor model. This is clearly different from breast cancer cell lines growing in 2D conditions that show a concordant expression of EMT genes as *VIM* and *SLUG* mirroring the expression of epithelial differentiation genes as *CDH1* or *EPCAM* (Kurt W. Kohn et al., 2014; K. W. Kohn et al., 2012). Other breast cancer decellularized scaffolds also showed an inverse correlation between EMT and differentiation markers, with an increased expression of *VIM*, *ZEB1* and *SNAIL* paralleled with a decreased *E‐cadherin* (Liu et al., 2019). In contrast, Dondajewska et al. (2018) observed differential expressions of different EMT markers in cells cultured in silk scaffolds in comparison to 2D cultures, similar to the behavior in the PDS cultures. An inhibition of *SLUG* and an elevated *VIM* expression were observed in this tumor model. We hypothesize that complex and more in vivo‐like mechanisms may regulate the EMT process in the PDS system and in some of the scaffold culture systems, generating expression discrepancies between the various EMT and differentiation markers. The complexity of the PDS platform, which includes the ECM structure and other tumor microenvironment‐associated proteins, may be crucial in the modulation of, for example, migration processes monitored by EMT‐associated genes.

The aim of this study was to establish how a human‐based microenvironment may affect drug responses. To determine the influence of the microenvironment on cancer cells, we used standardized breast cancer cell lines to repopulate the scaffolds, operating as a sensor and reporter for the cell–ECM interactions. However, the adaptability to the microenvironment will be dependent on specific cell line characteristics, as well as genetic abnormalities that might limit and potentially change the drug response (Gillet et al., 2011; Neve et al., 2006). In line with this, we observed general responses as well as varied drug effects in different cell lines cultured in the PDSs. Larger studies including more patient samples need to be performed to identify robust adaptation patterns. Moreover, it could be possible to repopulate the PDSs with the patient primary cancer cells to create a more complete model system that can be used to monitor patient‐specific responses to treatments. Nevertheless, due to the primary cells intrinsic heterogeneity, this approach would hide the tumor specific microenvironment influence achieved in this study when using a standardized cancer cell line as a reporter. Our model also presents the possibility to be complemented with additional stromal cells, such as macrophages and fibroblasts, which may play an important role in the signaling modulation between ECM and tumor cells and consequently in drug response (Dittmer & Leyh, 2015; Rijal & Li, 2016; Senthebane et al., 2017). However, the ECM has been produced and remodeled through dynamic interactions with all the cellular components of the tumor, including tumor and stromal cells, allowing PDSs to be used as adequate surrogates for the tumor microenvironment (Walker et al., 2018). In fact, exosome‐related proteins have been found in the PDSs proteomic composition (Landberg et al., 2020).

One more aspect to note in the PDS model is the variability between PDSs provided by different patients, as also observed in our previous studies (Landberg et al., 2020), and with other decellularized scaffolds (Pinto et al., 2017). This inter‐scaffold variability, which may be influenced by the original tumor subtype, was more evident for the PDSs cultured using T‐47D cells, suggesting that different cancer cell lines present different susceptibilities to the microenvironment. In addition, further studies should be performed to address if differences in the PDS composition or microstructure could be related to clinical parameters. The environment‐related information provided by the PDSs may be a complementary diagnostic tool and potentially provide prognostic information regarding clinical behavior and outcome as well as be predictive of treatment response.

## 5 CONCLUSION

This study provides an insight into the tumor microenvironment influence on chemotherapeutic response, using a physiologically relevant model provided directly from patients and consisting of actual human tumor ECM. Cancer cells cultured in this in vivo‐like PDS matrix adapted to the environment, increased their resistance and modified their response to different drug agents, providing a novel tool for preclinical drug studies. Furthermore, our results comparing a wide range of cell cultures and treatment conditions will be the foundation for further studies detailing the importance of variations in drug responses in PDS cultures, using more extensive tumor cohorts to potentially evaluate tumor response to chemotherapy in breast cancer patients.

## CONFLICT OF INTERESTS

The authors Maria Carmen Leiva, Elena Garre, Anna Gustafsson, Andreas Svanström, and Yalda Bogestål declare no potential conflict of interests. Göran Landberg and Anders Stålberg are board members and shareholders of Iscaff Pharma, and Anders Stålberg is a shareholder of TATAA Biocenter. The patient‐derived scaffold approach and data are covered in patent application. Patent applicants: Göran Landberg and Anders Stålberg; inventor: Joakim Håkansson; published with the international publication number: WO 2018/083231 A1.

## AUTHOR CONTRIBUTIONS

Conceived and designed the experiments: Maria Carmen Leiva, Elena Garre, Andreas Svanström and Göran Landberg. Conducted the experiments: Maria Carmen Leiva, Elena Garre, Anna Gustafsson and Andreas Svanström. Data collection, analysis, and interpretation: Maria Carmen Leiva, Elena Garre, Anna Gustafsson, Andreas Svanström, Göran Landberg and Anders Ståhlberg. Wrote the paper: Maria Carmen Leiva and Göran Landberg. Provided the 3D printed scaffolds: Yalda Bogestål and Joakim Håkansson. Critically reviewed, revised for intellectual content, and provided suggestions: Maria Carmen Leiva, Elena Garre, Anna Gustafsson, Andreas Svanström, Yalda Bogestål, Joakim Håkansson, Göran Landberg and Anders Ståhlberg. Supervised the project: Göran Landberg and Anders Ståhlberg. All authors reviewed the manuscript.

## Supporting information

Supporting information.

## Data Availability

The datasets are available from the corresponding author on reasonable request.
