# Supplementary material for: Breast cancer patient‐derived scaffolds as a tool to monitor chemotherapy responses in human tumor microenvironments
Source: J Cell Physiol. 2020 Dec 23;236(6):4709–24. doi: 10.1002/jcp.30191 (PMC8049042; doi:10.1002/jcp.30191)
Supplement: Supplementary file 1 — Supporting information. [file JCP-236-4709-s001.docx]

**Breast cancer patient-derived scaffolds as a tool to monitor chemotherapy responses in human tumor microenvironments**

**Running title: Patient-derived scaffolds and chemotherapy**

**Maria Carmen Leiva**^1^**, Elena Garre**^1^**, Anna Gustafsson**^1^**,Andreas Svanström**^1^**,Yalda Bogestål**^2^**,Joakim Håkansson**^1,2^**, Anders Ståhlberg**^1,3,4^**, Göran Landberg**^1^

^1^Department of Laboratory Medicine, Sahlgrenska Center for Cancer Research, Institute of Biomedicine, Sahlgrenska Academy, University of Gothenburg, Gothenburg, 413 90, Sweden.

^2^RISE Research Institutes of Sweden, Department of Biological Function, Box 857, 50115 Borås, Sweden

^3^Wallenberg Centre for Molecular and Translational Medicine, University of Gothenburg, Gothenburg, 413 90, Sweden.

^4^Department of Clinical Genetics and Genomics, Sahlgrenska University Hospital, Gothenburg, 413 90, Sweden.

SUPPLEMENTARY INFORMATION

**Table S1.**Histopathological characteristics of the beast tumors employed to obtain the patient-derived scaffolds used in this article. Neg = negative; Pos = positive; DCIS= ductal carcinoma *in situ*; LCIS= lobular carcinoma *in situ*, IDC = invasive ductal carcinoma; ILC = invasive lobular carcinoma; 5-FU = 5-fluorouracil; DOX = Doxorubicin; PTX = paclitaxel; PDS = patient-derived scaffold; 3DPS = 3D-printed scaffolds.

| **Tumor** | **ER (%)** | **PR (%)** | **HERCEP** | **KI67 (%)** | **Grade** | **Tumortype** | **Where it has been used in this article** |
| --- | --- | --- | --- | --- | --- | --- | --- |
| **1** | 0 | 0 | Neg | 58 | 3 | IDC | PDSs + MDA-MD-231 (PDS1) |
| **2** | 100 | 95 | Neg | 10 | 2 | LCIS +DCIS | PDSs + MDA-MB-231 (PDS2) |
| **3** | 100 | 100 | Neg | 55 | 3 | IDC | PDSs + MDA-MB-231 (PDS 3); PDSs +T-47D (PDS1) |
| **4** | 100 | 20 | Neg | 15 | 1 | IDC | PDSs + T-47D (PDS2) |
| **5** | 100 | 50 | Neg | 9 | 2 | IDC | PDSs + T-47D (PDS3) |
| **6** | 100 | 100 | Neg | 19 | 2 | ILC | PDSs+MCF7 treated with 5-FU and DOX above 50X and PTX above 100X (PDS1); Untreated (PDS1) |
| **7** | 100 | 5 | Neg | 23 | 2 | IDC | PDSs+MCF7 treated with 5-FU and DOX above 50X and PTX above 100X (PDS2); Untreated (PDS2) |
| **8** | 0 | 0 | Neg | 30 | 3 | IDC | PDSs+MCF7 treated with 5-FU and DOX above 50X and PTX above 100X (PDS3); Untreated (PDS3) |
| **9** | 100 | 10 | Neg | 32 | 3 | IDC + DCIS | PDSs + MCF7 treated with 5-FU, DOX and PTX at doses 5X and 10X (PDS1) |
| **10** | 100 | < 1 | Pos | 45 | 3 | IDC | PDSs + MCF7 treated with 5-FU, DOX and PTX at doses 5X and 10X (PDS2) |
| **11** | 100 | 50 | Neg | 43 | 2 | IDC | PDSs + MCF7 treated with 5-FU, DOX and PTX at doses 5X and 10X (PDS3) |
| **12** | 40 | 15 | Pos | 80 | 3 | IDC | PDSs + MCF7 treated with 5-FU, DOX and PTX at the IC_50_ concentration (PDS1) |
| **13** | 100 | <10 | Pos | 28 | 2 | IDC | PDSs + MCF7 treated with 5-FU, DOX and PTX at the IC_50_ concentration (PDS2) |
| **14** | 90 | 0 | Neg | 13 | 2 | IDC | PDSs + MCF7 treated with 5-FU, DOX and PTX at the IC_50_ concentration (PDS3) |
| **15** | 95 | 90 | Neg | 17 | 3 | IDC + DCIS | Comparison between PDSs and 3DPS |

**
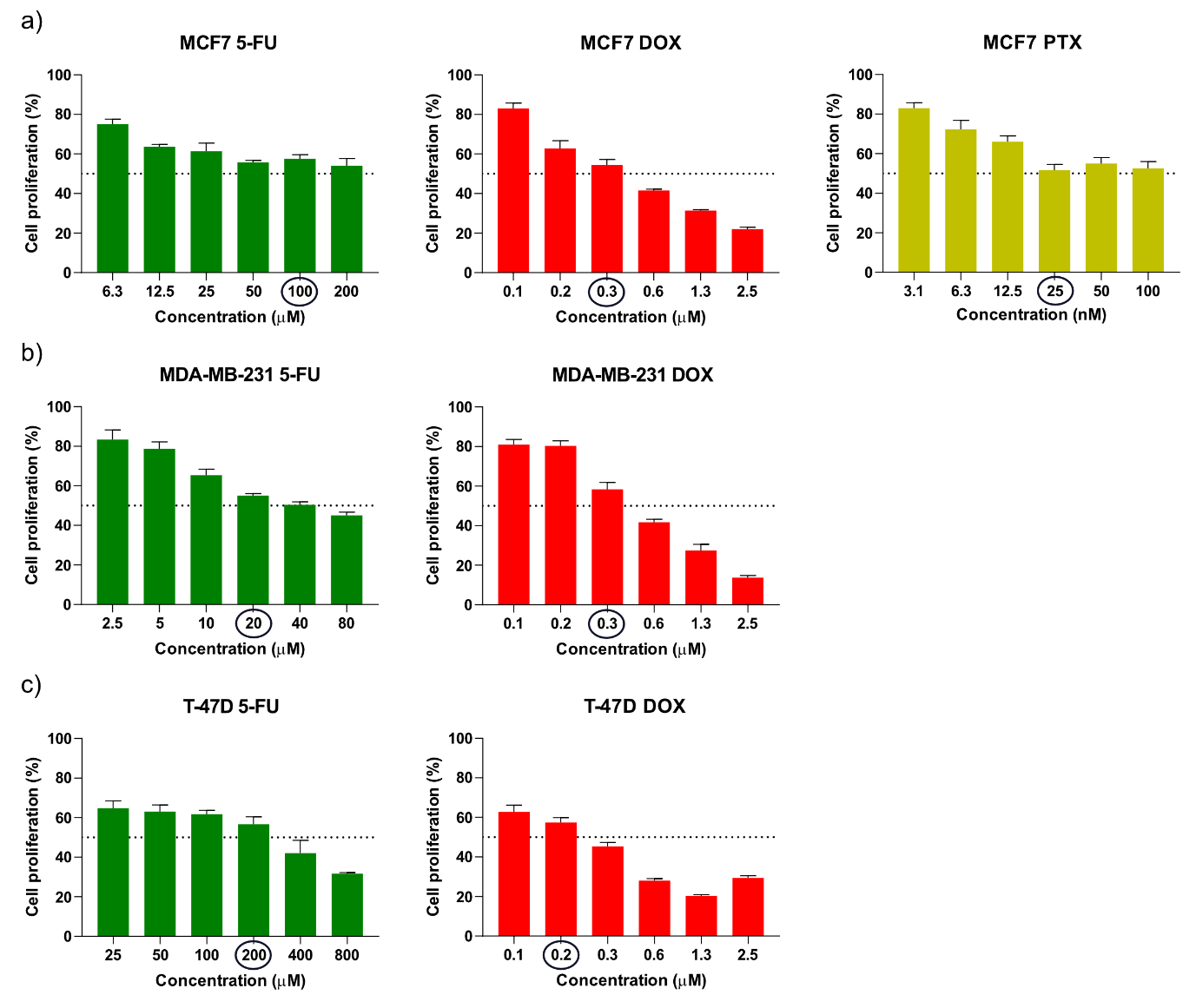
**

**Figure S1.**IC_50_ calculation in the 2D cultures. In order to establish suitable drug concentrations to test in patient-derived scaffolds, proliferation was assessed in (a) MCF7, (b) MDA-MB-231 and (c) T-47D cells following treatment with 5-fluorouracil (5-FU), doxorubicin (DOX) and paclitaxel (PTX; only in MCF7). Mean ± SD is shown, n = 3.Alamar blue metabolic assay was used and absorbance was measured before treatment and after 72 hours of treatment. The value after 72 hours was divided by the value before treatment to stablish the proliferation rate. Concentrations inhibiting proliferation by a 40-50%, which are highlighted in this figure, were chosen for further assays.

**Table S2.**List of genes and additional information.

| **Gene** | **OfficialName** | **Category** | **Ensembl Gene ID** | **Forward Primer [5'-3']** | **Reverse Primer [5'-3']** | **AmpliconLength [bp]** | **Intron spanning** |
| --- | --- | --- | --- | --- | --- | --- | --- |
| *ABCG2* | ATP-binding cassette, sub-family G (WHITE), member 2 | Breast cancer stem cell and multidrug resistance | ENSG00000118777 | GGTGGAGGCAAATCTTCGTTA | GAGTGCCCATCACAACATCA | 154 | Yes |
| *ALDH1A3* | Aldehydedehydrogenase 1 family, member A3 | Breast cancer stem cell | ENSG00000184254 | AAAAAGAGCGAATAGCACCG | GCATAGAGGGCGTTGTAGCA | 132 | Yes |
| *CCNA2* | Cyclin A2 | Proliferation | ENSG00000145386 | AAGACGAGACGGGTTGC | GGCTGTTTACTGTTTGCTTTCC | 89 | Yes |
| *CD24* | CD24 molecule | Epithelial/Differentiation | ENSG00000272398 | GCTCCTACCCACGCAGATT | GGTGGTGGCATTAGTTGGAT | 112 | No |
| *CD44* | CD44 molecule (Indian blood group) | Breast cancer stem cell | ENSG00000026508 | GAAGAAGGTGTGGGCAGAAGA | ACCATTTCCTGAGACTTGCTG | 112 | Yes |
| *CDH1* | Cadherin 1, type 1, E-cadherin (epithelial) | Epithelial/Differentiation | ENSG00000039068 | AGAGGACCAGGACTTTGACTTG | CAGAGAATCATAAGGCGGGG | 205 | Yes |
| *CDH2* | Cadherin 2, type 1, N-cadherin (neuronal) | EMT / metastasis | ENSG00000170558 | CATTATCAACCCCATCTCGG | ACTGTCCCATTCCAAACCTG | 198 | Yes |
| *EIF1* | eukaryotictranslation initiation factor 1 | Housekeeping / other | ENSG00000173812 | TCGTATGTCCGCTATCCAGA | TAAGGGTCTTCCTGCCGTTT | 137 | Yes |
| *EPCAM* | Epithelial cell adhesion molecule | Epithelial/Differentiation | ENSG00000119888 | CAGGAAGAATGTGTCTGTGAAAACT | TTCATTTCTGCCTTCATCACC | 152 | Yes |
| *FOSL1* | FOS-like antigen 1 | EMT / metastasis | ENSG00000175592 | GCAGGCGGAGACTGACAA | GGGGAAAGGGAGATACAAGG | 219 | Yes |
| *GAPDH* | glyceraldehyde-3-phosphate dehydrogenase | Housekeeping / other | ENSG00000111640 | AGTCAGCCGCATCTTCTTTT | CGCCCAATACGACCAAAT | 100 | Yes |
| *MKI67* | Antigen identified by monoclonal antibody Ki-67 | Proliferation | ENSG00000148773 | TGGGTCTGTTATTGATGAGCC | CATCAGGGTCAGAAGAGAAGC | 188 | Yes |
| *NANOG* | Nanoghomeobox | Stemness | ENSG00000111704 | CCTATGCCTGTGATTTGTGG | AAGTGGGTTGTTTGCCTTTG | 166 | Yes |
| *NEAT1* | Nuclearparaspeckleassembly transcript 1 | Stemness | ENSG00000245532 | GCCTTCTTGTGCGTTTCTCG | CCCTCCCAGCGTTTAGC | 158 | No |
| *POU5F1* | POU class 5 homeobox 1 | Stemness | ENSG00000204531 | CGAAAGAGAAAGCGAACCAG | AACCACACTCGGACCACATC | 146 | Yes |
| *RPS10* | Ribosomal protein S10 | Housekeeping / other | ENSG00000124614 | AGCCGCAGAGATGTTGATG | CCTCGGGACTTGAGAGACTG | 177 | Yes |
| *RPS26* | Ribosomal protein S26 | Housekeeping / other | ENSG00000197728 | GATGCGTGCCCAAGGAC | CAGGTCTAAATCGGGGTGG | 228 | Yes |
| *SLUG* | Snailfamilyzinc finger 2 | EMT / metastasis | ENSG00000019549 | GCCAAACTACAGCGAACTGG | AGGAGGTGTCAGATGGAGGA | 239 | Yes |
| *SNAI1* | Snailfamilyzinc finger 1 | EMT / metastasis | ENSG00000124216 | TAATCCAGAGTTTACCTTCCAGCA | AGCCTTTCCCACTGTCCTCA | 224 | Yes |
| *SOX2* | SRY (sex determining region Y)-box 2 | Stemness | ENSG00000181449 | ACACCAATCCCATCCACACT | CCTCCCCAGGTTTTCTCTGT | 117 | No |
| *TWIST* | Twist family bHLH transcription factor 1 | Stemness | ENST00000242261 | GGACAGTGATTCCCAGACGG | CATAGTGATGCCTTTCCTTTCAG | 188 | No |
| *VIM* | Vimentin | EMT / metastasis | ENSG00000026025 | CAGATGCGTGAAATGGAAGA | TGGAAGAGGCAGAGAAATCC | 222 | Yes |
| *YWHAZ* | Tyrosine 3-monooxygenase/tryptophan 5-monooxygenase activation protein, zeta | Housekeeping / other | ENSG00000164924 | ACGCCTCACTCCCGTTT | CTGGATGTTCTGCTGGCTC | 69 | Yes |
| DNA Spike II | TATAA Universal DNA Spike II | Qualitycontrol | NA | NA | NA | 286 | NA |

**Figure S2.**Comparison of the drug response between cells cultured in patient-derived scaffolds and 3D-printed scaffolds. Heatmaps illustrating gene expression changes in response to (a) doxorubicin (DOX) and (b) 5-fluorouracil (5-FU) in cells cultured in patient-derived scaffolds (PDS) and 3D-printed scaffolds (3DPS). *Significant differences between PDSs and 3DPS (P< 0.05, n=3).

**
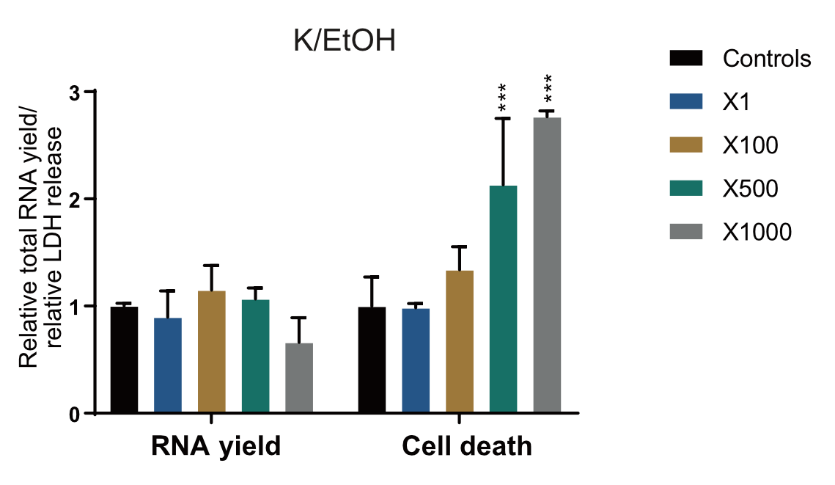
**

**Figure S3.** Paclitaxel solvent toxicity. Kolliphor/ethanoleffect on total RNA yield and LDH release, as surrogate measurements for cell number and cell death, respectively. Increased concentrations equivalent to paclitaxel concentrations used in Figure 6 are tested. Data arerelativeto untreated patient-derived scaffolds. Mean ± SD is shown, n = 3. Significant differences to the untreated controls are stated (***P<0.001).


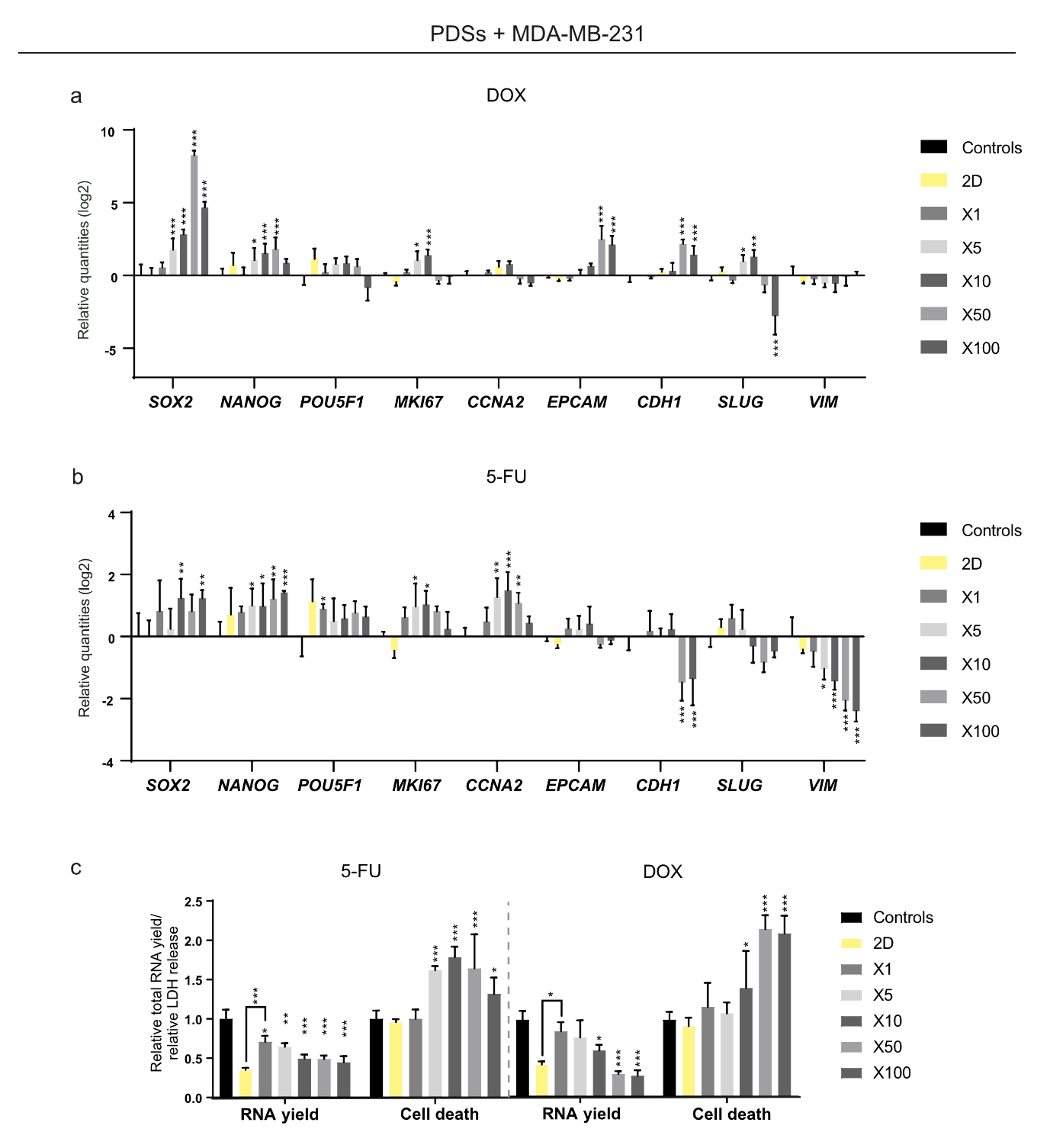


**Figure S4.** Analysis of MDA-MB-231 cells cultured in patient-derived scaffolds treated with doxorubicin and 5-fluorouracil. Modulation of gene expression following treatment with (a) doxorubicin (DOX) and (b) 5-fluorouracil (5-FU) at increasing concentrationsindicated as fold-change respect to the IC_50_ in 2D cultures. Data is related to gene expression in untreated patient-derived scaffolds (PDSs) (log2).(c) Drug effect on the total RNA yield andLDH release, as surrogate measurements for cell count and cell death, respectively, quantified at the same drug concentrations, in 5-FU- and DOX-treated PDS cultures. Relative quantities to untreated PDSs are represented.The drug effect in 2D cultures treated at the IC_50_ drug concentration is also illustrated (yellow) in all the figures, and in this case, data are normalized to untreated 2D samples. Mean ± SD is shown, n = 3. Differences in the expression of treated PDSs versus untreated controls, and 2D versus PDSs at the same concentration (X1) are stated (*P<0.05, **P<0.01, ***P<0.001).

**
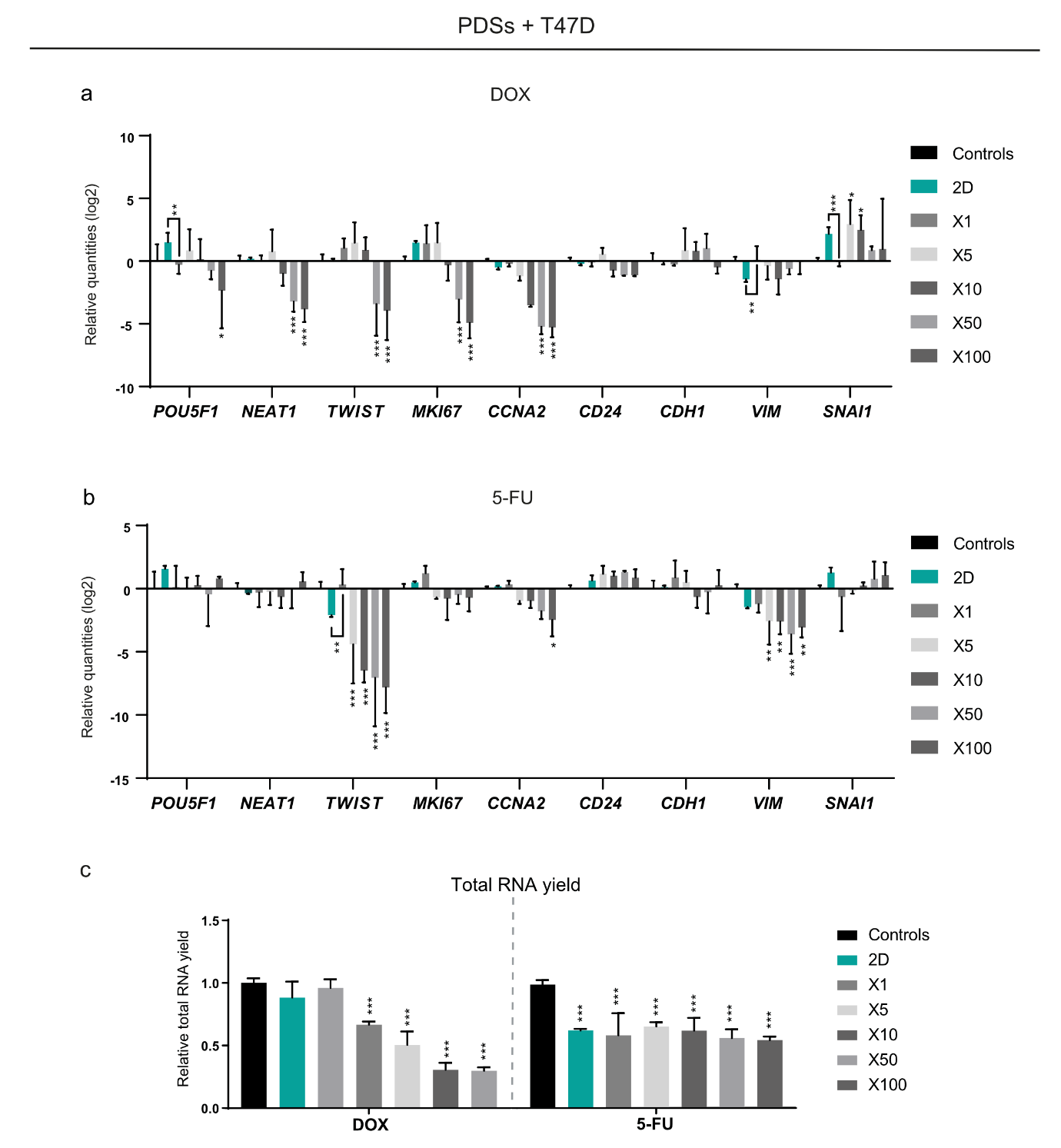
**

**Figure S5.** Analysis of T-47D cells cultured in patient-derived scaffolds treated with doxorubicin and 5-fluorouracil. Modulation of gene expression following treatment with (a) doxorubicin (DOX) and (b) 5-fluorouracil (5-FU) at increasing concentrationsindicated as fold-change respect to the IC_50_ in 2D cultures. Data is related to gene expression in untreated patient-derived scaffolds (PDSs) (log2). (c) Drug effect on the total RNA yield, as surrogate measurements for cell count, quantified at the same drug concentrations, in 5-FU- and DOX-treated PDS cultures. LDH assay could not be performed in this cell line. Relative quantities to untreated PDSs are represented.The drug effect in 2D cultures treated at the IC_50_ drug concentration is also illustrated (green) in all the figures,and in this case, data are normalized to untreated 2D samples. Mean ± SD is shown, n = 3. Differences in the expression of treated PDSs versus untreated controls, and 2D versus PDSs at the same concentration (X1) are stated (*P<0.05, **P<0.01, ***P<0.001).

**Table S3.**List of genes used in Fig. 8 to calculate the average expression in every family for the different cell lines. *ABCG2* was excluded from the CSC family due to its influence in drug resistance mechanisms.

|  | **MCF7** | **MDA-MB-231** | **T-47D** |
| --- | --- | --- | --- |
| **CSC** | *NANOG* | *NANOG* | *NEAT1* |
|  | *POU5F1* | *POU5F1* | *POU5F1* |
|  | *CD44* | *SOX2* | *TWIST* |
| **Proliferation** | *MKI67* | *MKI67* | *MKI67* |
|  | *CCNA2* | *CCNA2* | *CCNA2* |
| **EMT** | *VIM* | *VIM* | *VIM* |
|  | *SNAI1* | *SLUG* | *SNAI1* |
| **Differentiation** | *CD24* | *EPCAM* | *CD24* |
|  |  | *CDH1* | *CDH1* |
